# Varicella Vaccine

**DOI:** 10.1155/S1064744996000142

**Published:** 1996

**Authors:** Patrick Duff

**Affiliations:** Division of Maternal-Fetal Medicine University of Florida College of Medicine P.O. Box 100294 Gainesville FL 32610-0294 USA


Infectious Diseases in Obstetrics and Gynecology 4:63–65 (1996)
© 1996 Wiley-Liss, Inc.

### Varicella Vaccine

**Patrick Duff**
*Division of Maternal-Fetal Medicine, University of Florida, College of Medicine, Gainesville, FL*

Varicella is a common infectious disease that typically occurs in childhood. It is caused by the varicella-zoster (VZ) virus, a DNA herpesvirus that is transmitted by respiratory droplets or direct contact. Varicella is one of the most highly contagious of all viral infections. Approximately 80–90% of susceptible individuals become infected after exposure to an affected household member.[1]

In healthy children, varicella usually does not cause serious complications. However, immunocompromised children and otherwise healthy adults are at increased risk for life-threatening complications of varicella such as pneumonia, encephalitis, and disseminated infection. Although <10% of all cases of varicella occur in adults, this age group accounts for 25–30% of all varicella-related mortality.[2] In addition, when pregnant women contract varicella within the first 20 weeks of gestation, up to 2% of their neonates have evidence of varicella embryopathy.[3]

Following a primary varicella infection, the VZ virus may remain in a latent state for many years. Under certain conditions, such as stress or immunosuppression, the infection may be reactivated as herpes zoster. Although usually less severe than varicella, herpes zoster may also cause serious complications in some patients, particularly those with immunodeficiency disorders.

Because of the potentially serious sequelae of varicella in select groups of patients, researchers and clinicians have long been interested in the development of a vaccine to prevent the primary infection. In the latter part of 1995, the new varicella vaccine (Varivax®, Merck & Co., West Point, PA) finally was approved by the U.S. Food and Drug Administration for commercial marketing. The purpose of this article is to review the pharmacology, toxicity profile, clinical application, and cost of this important new immunobiologic agent.

### PHARMACOLOGY

The varicella vaccine was originally developed by Takahashi et al. in 1974. [1]The Oka/Merck strain used in the vaccine is attenuated by passage in human and embryonic guinea-pig cell cultures. The vaccine activates both the humoral and cell-mediated immune systems.

Children with leukemia were one of the original target groups for the vaccine. After investigational trials in normal children and adults, the vaccine was administered to leukemic children who were no longer receiving chemotherapy and, subsequently, to children who had been in remission for 1 year but still required maintenance chemotherapy. In these subjects, the vaccine has been highly immunogenic, producing seroconversion in 90% and 97% of patients after 1 and 2 doses, respectively.[1,4] In healthy children, 1–12 years old, a single dose of vaccine results in seroconversion in approximately 97%. In healthy adolescents and adults, the rate of seroconversion is approximately 80% after 1 dose and 90–95% after 2 doses.[1,4,5]

Several investigators have demonstrated that immunity following vaccination is long lasting. Asano et al.[6] evaluated 25 Japanese children who received one of the earliest vaccines prepared from the Oka strain and found persistent antibody titers and cellular immune responses 20 years after vaccination. Watson and colleagues[7] followed 214 American children for 6 years after vaccination and documented persistent antibody in 95%. Moreover, 94% had evidence of VZ-specific lymphocyte proliferation, indicating appropriate activation of cell-mediated immunity in response to the varicella virus. Ninety-

Address correspondence/reprint requests to Dr. Patrick Duff, Division of Maternal-Fetal Medicine, University of Florida, College of Medicine, P.O. Box 100294, Gainesville, FL 32610-0294.

Received June 10, 1996
Accepted June 18, 1996

one percent of children had persistence of *both* humoral and cell-mediated immunity. In a subsequent investigation, Gershon et al.[5] followed 40 adults for 7–13 years after vaccination and noted persistent antibody in 82%.

The varicella vaccine is highly effective, but not perfectly protective, in preventing natural infection. In children with leukemia, the vaccine has reduced the rate of transmission of chickenpox from 80–90% to 15–20%.[1,4] In addition, leukemic children who develop varicella despite immunization have much milder infections than would be anticipated with natural infection. In normal children, the protective efficacy of the vaccine is even higher. Weibel et al.[8] conducted a double-blind, placebo-controlled trial of the vaccine in 956 children between ages 1 and 14 years. None of the 468 children who received the vaccine developed varicella during the 9-month period of observation after vaccination. Thirty-eight of the 446 placebo recipients became infected ($P < 10^{-9}$). In adolescents and adults, the protective efficacy of the vaccine is approximately 70–75%.[1,4]

### ADVERSE EFFECTS

The frequency of serious adverse effects following the administration of the varicella vaccine is relatively low. Approximately 10–15% of vaccinees experience fever, 5–10% develop reactions at the site of injection, and 3–5% develop maculopapular or vesicular eruptions. Fewer than 1% of vaccine recipients have fatigue, cough, vomiting, diarrhea, headache, malaise, lymphadenopathy, arthralgias, pruritus, or febrile seizures. Adolescents and adults are more likely to have side effects than children aged 1–12 years. Vaccine recipients should be cautioned against taking salicylates for control of the above reactions because of the possibility of developing Reye's syndrome.[1,4,9]

Recipients of the varicella vaccine, particularly leukemic children, who develop skin rashes may transmit the virus to susceptible close contacts.[10] The frequency of transmission is approximately 10%, which is much lower than the transmission rate with natural viral infection. Patients who do not develop rashes apparently do not transmit the virus to susceptible contacts. Vaccine recipients, even those who are immunocompromised, do not have an increased frequency of herpes zoster compared with individuals who are infected with the wild virus.[1,4,9] In fact, the subsequent incidence of herpes zoster in vaccinees may be decreased.[11]

### CLINICAL APPLICATION

The varicella vaccine is indicated for all susceptible, immunocompetent individuals older than 1 year. Prior to the administration of the vaccine, the patient's immune status should be verified by a clearly documented medical history or a confirmatory VZ serology. A child between ages 1 and 12 years should receive one 0.5 ml subcutaneous dose of the vaccine. An adolescent or adult requires 2 vaccinations, 4–8 weeks apart. The vaccine can be given simultaneously with the measles-mumps-rubella (MMR) vaccine, provided that separate syringes and injection sites are used.[8,9]

Approximately 6–8 weeks after vaccination, a serologic test should be performed to document seroconversion. A child between ages 1 and 12 years who fails to seroconvert after a single injection may subsequently respond to a second vaccination. Individuals >12 years of age who do not seroconvert after 2 doses should be advised that they remain susceptible to natural varicella infection.

As noted previously, the original target group for the varicella vaccine was children with leukemia. Even in this population, the vaccine has been highly immunogenic and remarkably safe. Although immunodeficiency is listed as a contraindication to use of the vaccine, vaccination still may be considered in immunocompromised patients under carefully designed treatment protocols.

### COST

The wholesale cost of the varicella vaccine is approximately $40 per dose.[4] The vaccine must be stored in a freezer and then reconstituted and administered within 30 min of thawing. Accordingly, the actual charge to the patient for the vaccine may be slightly above $40 because of the added costs of preparation and administration.

### CONCLUSIONS

The varicella vaccine is a live attenuated vaccine that is approximately 70–80% effective in preventing, or attenuating, the course of a natural varicella infection. A child between ages 1 and 12 years should receive 1 subcutaneous dose of the vaccine. An adolescent or adult requires 2 doses, administered 4–8 weeks apart. The principal adverse effects associated with the administration of the vaccine are pain and tenderness at the injection site and a sparse, maculopapular or vesicular rash. The varicella vaccine should not be given to pregnant women or to patients who have received systemic corticosteroids within the month prior to vaccination. It also should not be routinely administered to immunodeficient patients except under a strictly supervised research protocol.

### REFERENCES

1. Gershon AA: Live attenuated varicella vaccine. J Infect Dis 152:859–862, 1985.
2. Chapman S, Duff P: Varicella in pregnancy. Semin Perinatol 17:403–409, 1993.
3. Enders G, Miller E, Cradock-Watson J, Bolley I, Ridehalgh M: Consequences of varicella and herpes zoster in pregnancy: Prospective study of 1,739 cases. Lancet 343:1547–1550, 1994.
4. Varicella vaccine. Med Lett 37:55–57, 1995.
5. Gershon AA, Steinberg SP, LaRussa P, Ferrara A, Hammerschlag M, Gelb L, et al.: Immunization of healthy adults with live attenuated varicella vaccine. J Infect Dis 158:132–137, 1988.
6. Asano Y, Suga S, Yoshikawa T, Kobayashi I, Yazaki T, Shibata M, et al.: Experience and reason: Twenty year follow-up of protective immunity of the Oka strain live varicella vaccine. Pediatrics 94:524, 1994.
7. Watson B, Gupta R, Randall T, Starr S: Persistence of cell-mediated and humoral responses in healthy children immunized with live attenuated varicella vaccine. J Infect Dis 169:197–199, 1994.
8. Weibel RE, Neff BJ, Kuter BJ, Guess HA, Rothenberger CA, Fitzgerald AJ, et al.: Live attenuated varicella vaccine. Efficacy trial in healthy children. N Engl J Med 310:1409–1415, 1984.
9. Greco H: Prevention of chickenpox in reproductive-age women. Prim Care Update 2:221–223, 1995.
10. Hughes P, LaRussa P, Pearce JM, Lepow M, Steinberg S, Gershon A: Transmission of varicella-zoster virus from a vaccinee with leukemia, demonstrated by polymerase chain reaction. J Pediatr 124:932–935, 1994.
11. Hardy IB, Gershon AA, Larussa PS, Steinberg SP: The incidence of zoster after immunization with live attenuated varicella vaccine: A study in children with leukemia. N Engl J Med 325:1545–1550, 1991.
